# Pulmonary nocardiosis masquerading renascence of tuberculosis in an immunocompetent host: a case report from Nepal

**DOI:** 10.1186/s13104-018-3604-2

**Published:** 2018-07-17

**Authors:** Priyatam Khadka, Ramesh Bahadur Basnet, Basista Parsad Rijal, Jeevan Bahadur Sherchand

**Affiliations:** 1 Tribhuvan University Teaching Hospital, Kathmandu, Nepal; 2 Trichandra Multiple Campus, Tribhuvan University, Ghantaghar, Kathmandu, Nepal

**Keywords:** Immunocompetent host, Pulmonary nocardiosis, Tuberculosis

## Abstract

**Background:**

Pulmonary nocardiosis is an opportunistic infection in an immunocompromised patient; however, often neglected in the immunocompetent patient from the diagnosis considerations.

**Case presentations:**

We describe a case of pulmonary nocardiosis masquerading renascence of tuberculosis, in a 51-years-Nepali farmer. After a 6 month of presumed successful antitubercular therapy; the patient develops the clinical presentations and radiological features showing similarities with that of tuberculosis and malignancy. MTB complex was not detected with Xpert MTB/RIF assay and cytological examinations were negative for the malignant cells, however. The Ziehl–Neelsen staining of the broncho-alveolar-lavage revealed acid-fast, thin branching filamentous organisms suggestive *Nocardia* spp. Further, identifications and susceptibility pattern against recommended antibiotics were assessed as per the CLSI guidelines. The case was then, subsequently, diagnosed as pulmonary nocardiosis. Trimethoprim–sulfamethoxazole was prescribed for 12 months. The patient underwent progressive changes and no relapse was noted in a periodic follow-up.

**Conclusions:**

This case underscores that pulmonary nocardiosis requires diagnostic considerations, regardless of a patient’s immunologic status and other mimicking infections.

## Background

Pulmonary nocardiosis often imitates pulmonary tuberculosis in both clinical presentations and radiological characteristics [1]. Consequently, the diagnosis of nocardiosis can be overlooked and treated empirically with anti-tuberculosis regimens; when in fact pulmonary nocardiosis should have been treated [2]. The condition is more common in developing countries where the diagnosis is rely based on these findings [3, 4]. Pulmonary nocardiosis is an opportunistic infection in an immunocompromised patient; however, often neglected in the immunocompetent patient from the diagnosis considerations [5]. Herein, we report a case of pulmonary nocardiosis in an immunocompetent host featuring as a relapse case of tuberculosis.

## Case presentations

A 51-year-old man presented in Tribhuvan University teaching hospital (TUTH); with chief complain of fever, severe chest pain and dry cough for 3 weeks. The patient denied any history of diabetes, hypertension, and asthma. Neither breathlessness nor hemoptysis was reported. At that time, he underwent chest radiography and sputum examination. The posterior–anterior (PA) view of chest x-ray was suggestive of pulmonary tuberculosis. However, no acid-fast bacilli found in two consecutive sputum samples. Later on, the case diagnosed as pulmonary tuberculosis (rifampicin sensitive) with Xpert MT/RIF Assay. The patient received HRZE for 2 months, followed by HR for 4 months as followed by antitubercular therapy in a government-run tuberculosis center (DOTS). After a subsequent antitubercular therapy for 6 months, the symptoms subsided, chest Xray showed a decreased size of opacity and no AFB seen on smear microscopy. Hence, declared cured and remained asymptomatic succeeding 4 months.

**Fig. 1:**
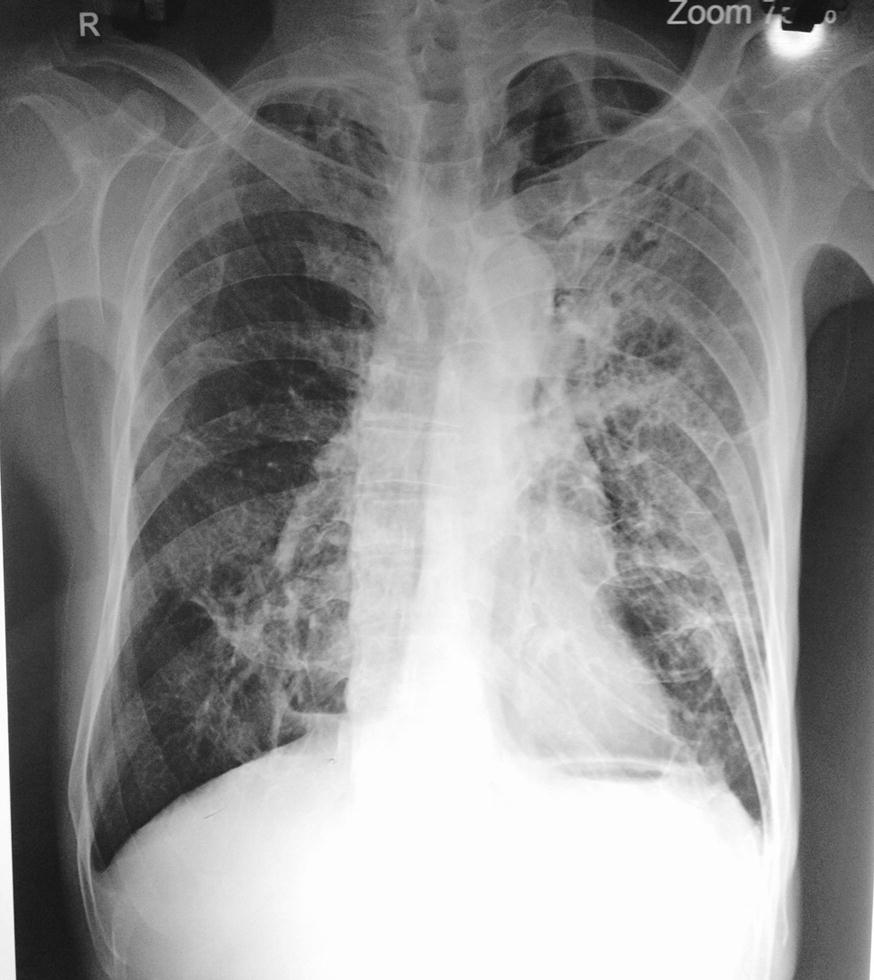
posterior–anterior (PA) view chest x-ray: left perihilar opacity with fibrotic changes

Nearly after 4 months of presumed successful treatment, the sequel resumed with similar clinical presentations but this time with worsened cough including hemoptysis. On posterior–anterior (PA) view chest x-ray, left perihilar opacity with fibrotic changes was seen (Fig. 1). Based on clinical manifestation, radiological features and AFB positive reported on smear microscopy, relapse of pulmonary tuberculosis was assumed. Further, GeneXpert test was done to rule out MDR TB or mutation on genome if present; nevertheless, no MTB complex was detected. On general examination, he was conscious, oriented and well nourished; no clubbing and cervical lymphadenopathy noted.

## Investigation

A complete blood count reveals: Haemoglobin: 10.0 gm/dl; white blood cell (WBC) count: 14,500/mm^3^; platelets 150 × 109/L; neutrophils: 85%; lymphocytes: 8%; monocytes: 5%; eosinophils: 2; ESR: 45 mm in the first hour. A peripheral blood smear for haemoparasite was negative. Serological marker (HIV/HBsAg/HCV) and Widal test were negative. Similarly, renal function, liver function, blood glucose were normal.

**Fig. 2:**
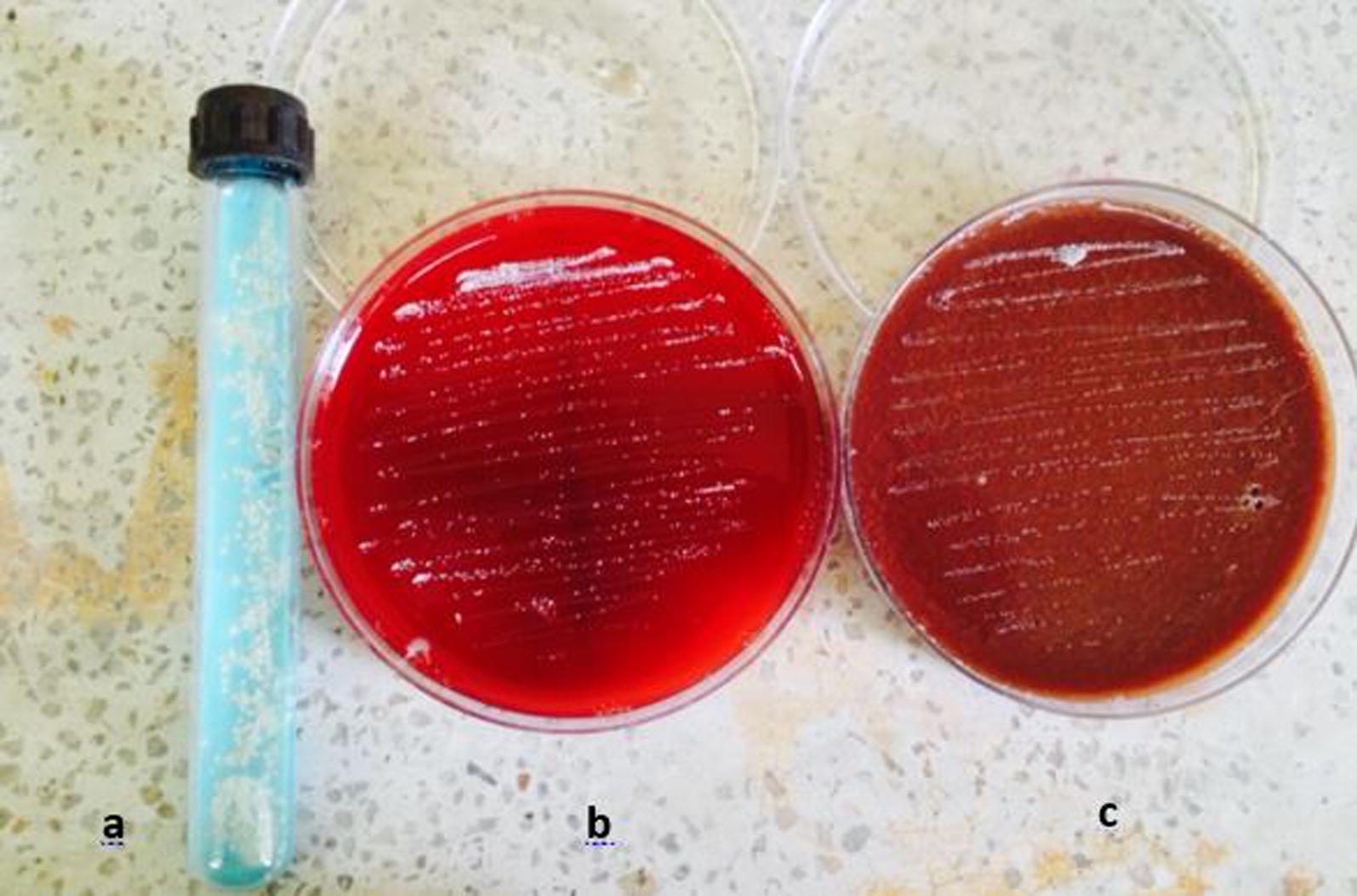
AFB staining: partially acid-fast branching rod suggestive *Nocardia species* on modified. Kinyounstain (×1000 orginal magnification)

**Fig. 3:**
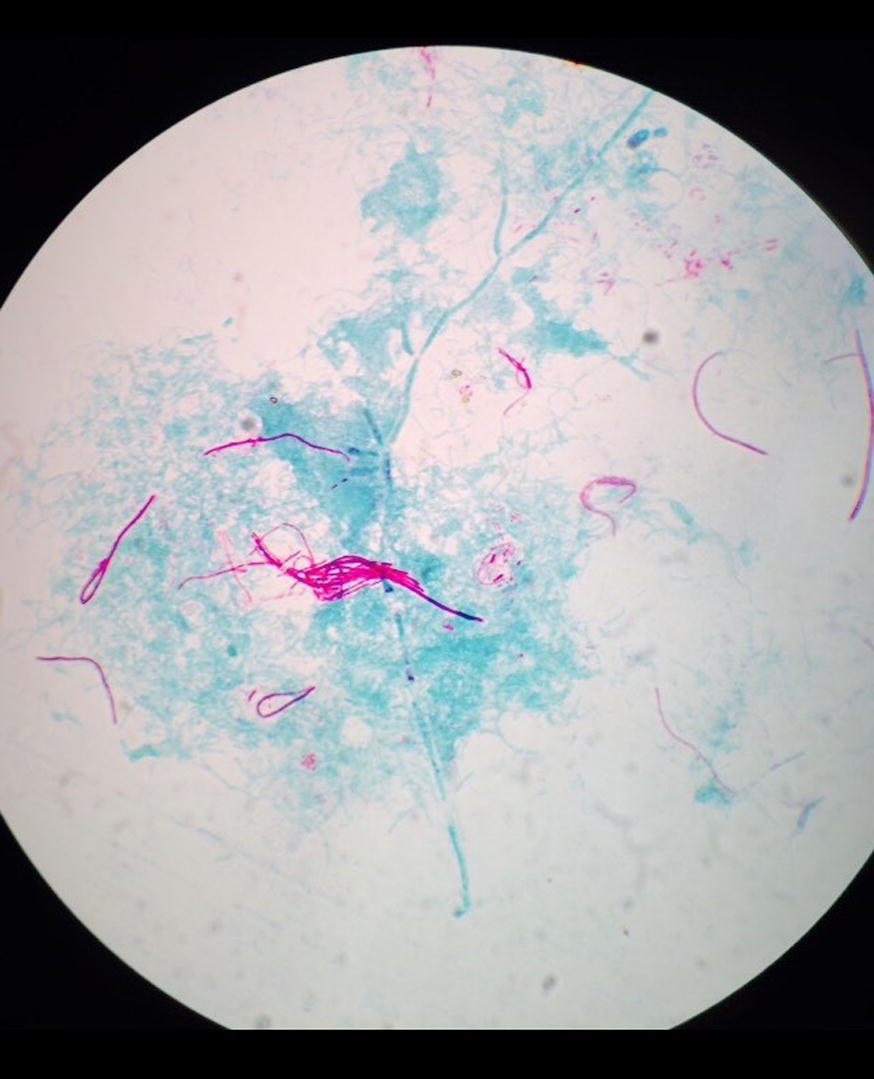
Colonial morphology of *Nocardia species* on **a** LJ media; **b** Blood Agar; **c** Chocolate agar: whitish chalky adherent colonies of *Nocardia species*

Sputum grew non-significant bacterial, while Xpert MTB/RIF Assay detects no MTB complex. Since the patient was unable to expectorate the patient underwent bronchoscopy; broncho-alveolar-lavage was aspirated then sent for cytological and microbiological analysis. No associated malignancy reported on histopathological examinations. However, Ziehl Neelsen staining of the broncho-alveolar-lavage revealed acid-fast thin branching filamentous organisms suggestive of nocardiosis (Fig. 2). The species are grown on Blood agar, Chocolate agar, and LJ media after 72 h of incubations with a chalky white appearance on blood agar, chocolate agar and LJ media (Fig. 3).

Further, identification of the isolate was done with standard microbiological culture methods recommended by American Society for Microbiology [6]. In brief, colony morphology (chalky, matt, dry, crumbly, adherent or velvety in appearances; 0.5–1.0 mm in diameter with fine intertwining, branching filaments with delicate aerial hyphae were seen); in-house set of biochemical test: catalase (positive), Urea hydrolysis test (positive), Bile esculin (positive), Acid production on rhamnose (positive), Acid production sorbitol (positive), Simmon’s citrate (negative), Nitrate reduction (negative), no gelatin liquefaction and hydrolysis of casein, tyrosine, and xanthine seen; varied incubation temperature of the isolates (under incubation with temperature variation at 46 and 10 °C, no growth was seen at 10 °C but seen at 46 °C). The antibiotic sensitivity test was performed by Kirby Bauer disc diffusion method following CLSI guidelines [7]. The isolate was sensitive to Amikacin, Imipenem, Cotrimoxazole, while Amoxicillin, Ceftriaxone, and Cefotaxime were found intermediate.

## Treatment

The patient was treated with BACTRIM-DS (PO × BD), 2 double strength tablets each containing 800 mg sulfamethoxazole and 160 mg trimethoprim. Fever and cough subsided within 6 days of antimicrobial therapy.

## Outcomes and follow-up

The patient recovered well, on medication with BACTRIM-DS (PO × BD), 2 double strength tablets each containing 800 mg sulfamethoxazole and 160 mg trimethoprim and was prescribed for 3 months then to follow. The same medication was continued for 12 months, the patient underwent progressive changes and no relapse was noted. He is under regular follow-up, from then and we found him asymptomatic, with limited side effects—possibly due to prolonged antimicrobial therapy.

## Discussion and conclusion

Nocardiosis is an opportunistic infection profoundly affects the immunocompromised patient; however, often neglected in an immunocompetent patient from the diagnosis considerations [5]. It has been reviewed in a study, that 15% of patients with nocardiosis do not have a predisposing immunosuppressive condition and the figure ranges from 10 to 25% on other studies [8]. This case underscores, regardless of a patient’s immunologic status, the isolation of *Nocardia* from the respiratory tract or other body sources should not be regarded as a contaminant or commensal organism [9].

The infection result due to inhalation (pulmonary nocardiosis-pneumonia, lung abscess, and cavitary lesions) or contact with the bacteria via a cut or abraded skin (cutaneous nocardiosis-cellulitis, ulcers); and possibly metastasizes haematogenously into distant organs system (lungs, central nervous system, eyes, kidneys, skin, subcutaneous tissue and bone) [10]. Relating to our case, probably the inhalation of the pathogen attribute for the progressive pulmonary nocardiosis.

The clinical syndromes vary and range from pulmonary, disseminated, cutaneous form involving eyes, kidneys, skin, bone, and CNS. The lungs, however, are the most common site of involvement and are affected in 70% of all cases of nocardiosis [11]. The clinical presentations of pulmonary nocardiosis are nonspecific and erratic with a chronic course resulting diagnostic stalemate. Consequently, delay and the high propensity of misdiagnosis may be attributed, as the clinical features—fever, cough, breathlessness, hemoptysis, and weight loss—mimic pulmonary tuberculosis, invasive fungal disease, community-acquired pneumonia, and lungs cancer [12].

In a diagnostic perspective of pulmonary nocardiosis, the chest radiographic indices are pleomorphic and nonspecific. The radiological picture revealing, consolidations and large irregular nodules, often cavitary, are most common; however, nodules, masses, and interstitial patterns may also be seen [13]. The case we present found obscured apart, with similar clinical presentations and radiological features show semblance to pulmonary tuberculosis.

The identification of the isolate was done with standard microbiological culture methods recommended by American Society for Microbiology based on phenotypic characteristics, biochemical interpretations, and varied incubation temperatures since molecular analysis and sequencing was not accessible in our laboratory settings. The microbiological diagnosis, however, is often difficult owing to the longer incubation periods to cultivate the pathogen in vitro and variable characteristics on gram staining, acid-fast-stain negative as if positive, also in the distorted branching forms mimicking *Mycobacterium tuberculosis* [2]. Henceforward, PCR and molecular-based diagnostic tools are of utmost importance in an early diagnosis of the nocardiosis. Therefore, a high index of clinical suspicion together with close collaboration with microbiological and radiological interpretations allows the more precise diagnosis to initiate appropriate therapy.

The implicated clinical management, nevertheless, of nocardiosis is the stereotaxic aspiration or surgical resection, and a course of appropriate antibiotics therapy for several months [14]. Unfolding of our case, the patient was treated with trimethoprim–sulfamethoxazole which undergoes a better prognosis of the condition. Furthermore, to limit the possibility of late relapse of nocardiosis the treatment regimen was extended to 12 months. The alternative parenteral antimicrobial therapies of carbapenems (imipenem or meropenem, but not ertapenem), third-generation cephalosporins (cefotaxime or ceftriaxone), and amikacin, alone or in combination were presumed unnecessary to include in regimen, though recommended by some author [15, 16]. Nonetheless, the likelihood of late recurrent nocardiosis could be a pitfall to the successful outcomes [12, 17]. Hereafter, clinical management along with the consistent follow up is crucial in the elimination of the infection.

However, the adverse reactions, owing to prolonged and high-dose TMP-SMX therapy, are frequent including: myelosuppression, hepatoxicity, immune hypersensitivity reactions and renal insufficiency [14]. Nonetheless, no such complications were found to be associated with our case. As a minimal side effect, the patient complained a few episodes of nausea, vomiting, and anorexia during 12 months of medications.

The anti-tubercular drugs have their own spectrum of hematological toxicity and blood cell abnormalities along with drug-induced syndrome—hemolytic anemia, methemoglobinemia, red cell aplasia, sideroblastic anemia, megaloblastic anemia, polycythemia and aplastic anemia—affecting natural immune functions to acquire the pulmonary nocardiosis [18]. Relating to our case, possibly the antitubercular drugs could be a potent risk factor for the successive pulmonary nocardiosis.

The burden and case associated with pulmonary nocardiosis—particularly in an immunocompetent host—in developing countries like Nepal are undetermined and unkempt. The exoticism with the case, nonspecific or lack of pathognomonic clinical presentation, diagnostic intricacies, and lack of systematic reporting impede further workup for nocardiosis [19, 14]. To our knowledge, this is the first case of pulmonary nocardiosis of different clinical type—masquerading renascence of pulmonary tuberculosis—in an immunocompetent host from Nepal, although nocardiosis in the immunocompromised has been reported elsewhere.

This case underscores that pulmonary nocardiosis requires diagnostic considerations if patients’ condition exacerbates despite optimum antitubercular therapy or in presumed relapse cases. Regardless of a patient’s immunologic status, the isolation of *Nocardia* sps from the respiratory tract or other body sources, should not be overlooked as a contaminant or commensal organism.

## Abbreviations

AFB: acid fast bacilli
CLSI: Clinical & Laboratory Standards Institute
CNS: central nervous system
DOTS: directly observed therapy short-course
ESR: erythrocyte sedimentation rate
HIV: human immune defeciency virus
HBsAg: hepatitis B surface antigen
HCV: hepatitis C virus
LJ: Löwenstein–Jensen medium
MDR: multi drug resistant
MTB: *Mycobacterium tuberculosis*
PCR: polymerase chain reaction
TUTH: Tribhuvan University Teaching Hospital
