# Correction to: Pulmonary nocardiosis masquerading renascence of tuberculosis in an immunocompetent host: a case report from Nepal

**DOI:** 10.1186/s13104-018-3619-8

**Published:** 2018-07-25

**Authors:** Priyatam Khadka, Ramesh Bahadur Basnet, Basista Parsad Rijal, Jeevan Bahadur Sherchand

**Affiliations:** 1 Tribhuvan University Teaching Hospital, Kathmandu, Nepal; 2 Trichandra Multiple Campus, Tribhuvan University, Ghantaghar, Kathmandu, Nepal

## Correction to: BMC Res Notes (2018) 11:488 10.1186/s13104-018-3604-2

Following publication of the original article [1], a typesetting mistake is reported. The captions of Figs. 2 and 3 were interchanged. The correct combination of the figures and captions (Figs. 2, 3) is given in this Correction and the original article has been updated.

**Fig. 2:**
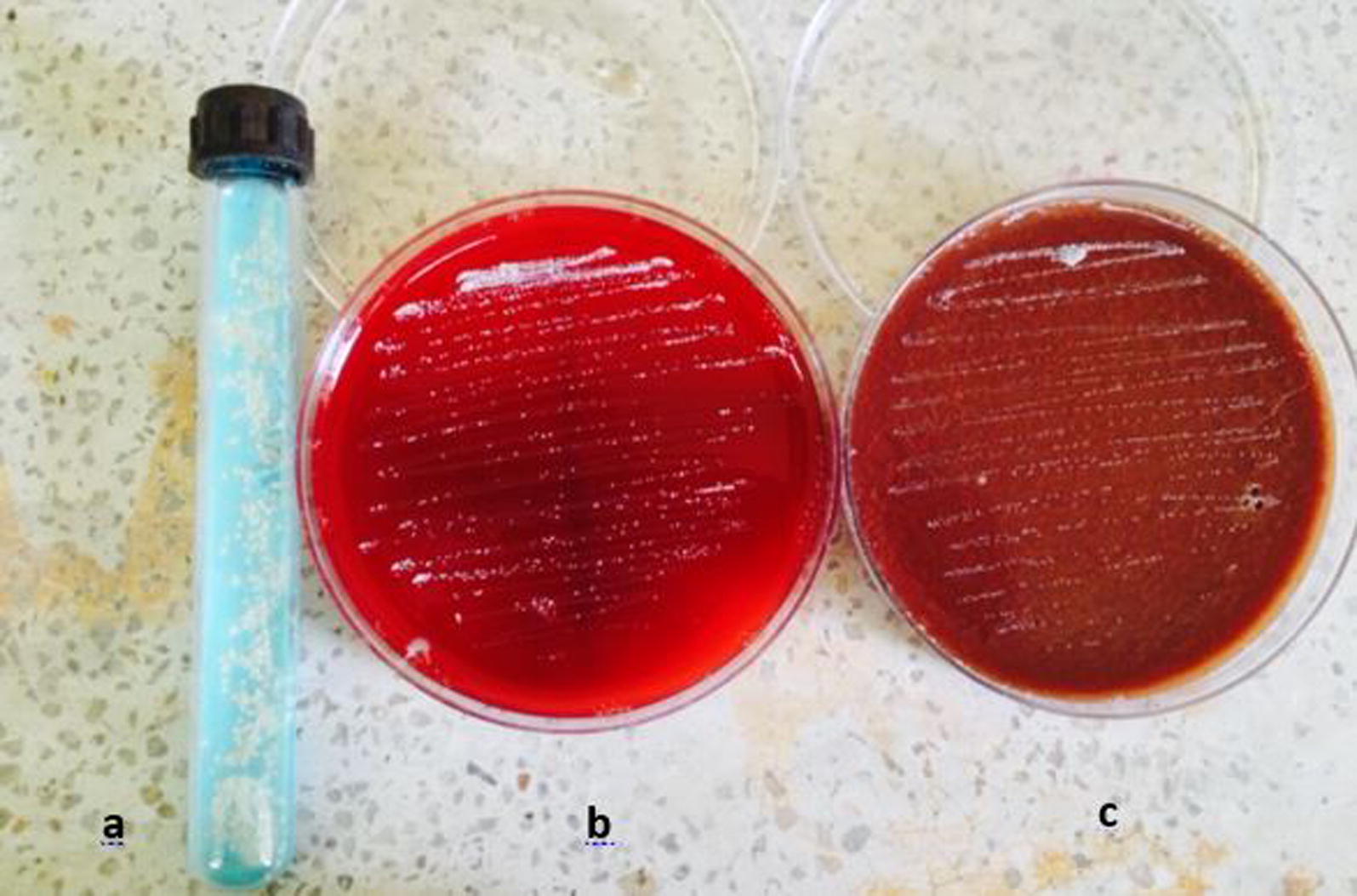
Colonial morphology of *Nocardia* species on **a** LJ media; **b** blood agar; **c** chocolate agar: whitish chalky adherent colonies of *Nocardia* species

**Fig. 3:**
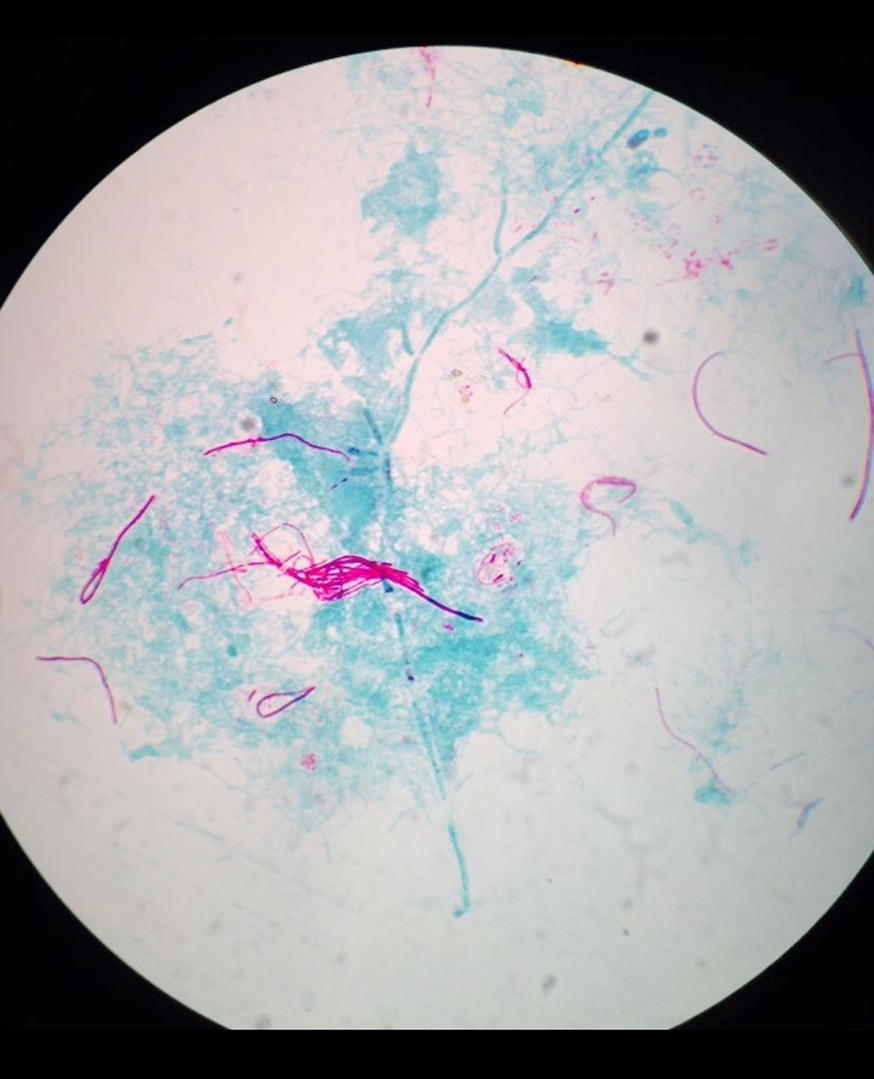
AFB staining: partially acid-fast branching rod suggestive *Nocardia* species on modified. Kinyounstain (×1000 original magnification)

The publisher apologizes to the authors and readers for the inconvenience.
